# A functional AT/G polymorphism in the 5′-untranslated region of *SETDB2* in the IgE locus on human chromosome 13q14

**DOI:** 10.1038/gene.2015.36

**Published:** 2015-09-17

**Authors:** R J Holt, C Vandiedonck, S A Willis-Owen, J C Knight, W O Cookson, M F Moffatt, Y Zhang

**Affiliations:** 1 Wellcome Trust Centre for Human Genetics, University of Oxford, Oxford, UK; 2 Molecular Genomics and Genetics Group, National Heart and Lung Institute, Imperial College London, London, UK

## Abstract

The immunoglobulin E (IgE)-associated locus on human chromosome 13q14 influencing asthma-related traits contains the genes *PHF11* and *SETDB2*. *SETDB2* is located in the same linkage disequilibrium region as *PHF11* and polymorphisms within *SETDB2* have been shown to associate with total serum IgE levels. In this report, we sequenced the 15 exons of *SETDB2* and identified a single previously ungenotyped mutation (AT/G, *rs386770867*) in the 5′-untranslated region of the gene. The polymorphism was found to be significantly associated with serum IgE levels in our asthma cohort (*P*=0.0012). Electrophoretic mobility shift assays revealed that the transcription factor Ying Yang 1 binds to the AT allele, whereas SRY (Sex determining Region Y) binds to the G allele. Allele-specific transcription analysis (allelotyping) was performed in 35 individuals heterozygous for *rs386770867* from a panel of 200 British families ascertained through probands with severe stage 3 asthma. The AT allele was found to be significantly overexpressed in these individuals (*P*=1.26 × 10^−21^). A dual-luciferase assay with the pGL3 luciferase reporter gene showed that the AT allele significantly affects transcriptional activities. Our results indicate that the IgE-associated AT/G polymorphism (*rs386770867*) regulates transcription of *SETDB2*.

## Introduction

Asthma is a complex disease underpinned by a combination of genetic and environmental factors. Positional cloning and genome-wide association studies have resulted in the identification of a number of loci that influence asthma and asthma-related traits such as total serum immunoglobulin E (IgE).^1^ An understanding of the function of the genes within these loci as well as their regulation will provide important insights into their roles in disease.

Previously, we have reported a highly significant genetic association between a locus on human chromosome 13 and total serum IgE.^2^ The locus contains the genes *PHF11* and *SETDB2*. Both genes are in the same linkage disequilibrium (LD) block and are separated by a mere 4 kb. The association between single-nucleotide polymorphisms in *PHF11* and asthma, or asthma-associated traits, has been investigated in many subsequent studies, with reports confirming the association in a number of ethnic populations.^3, 4, 5^ It is notable, however, that two studies have failed to replicate the original finding^6, 7^ potentially due to true differences in the effect of *PHF11* variants between groups (e.g. due to variation in environmental exposures or through epistatic or gene–environment interactions) and variance in the patterns of LD between typed and true causative variants.

*SETDB2* is transcribed in the same direction as *PHF11* and cotranscripts extending from *SETDB2* to *PHF11* exist in many human tissues.^2^ Owing to the small intervening distance and the LD structure between these genes, it is not currently possible to delineate the relative roles of *SETDB2* and *PHF11* in asthma and its associated traits. *SETDB2* has a methyl-CpG-binding domain and SET domain that modulates gene expression epigenetically through histone H3 lysine methylation. Abnormal histone methylation has been found in many diseases including asthma.^8^ In this report, we sequenced the 15 exons of *SETDB2* to discover new polymorphisms that may underlie asthma-associated traits. We identified an AT/G mutation (*rs386770867*), comprising both an insertion and substitution at the same locus, positioned in the 5′-untranslated region (UTR) of *SETDB2*. We genotyped this variant in our family-based cohorts of asthma and then investigated its role in the regulation of *SETDB2* using electrophoretic mobility shift assays (EMSAs), alleotyping and dual reporter gene assay analysis.

## Results

### *SETDB2* sequencing and genotyping

**Figure 1:**
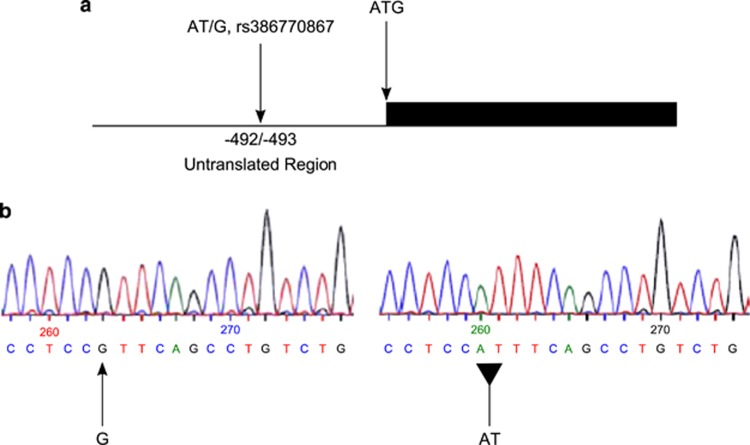
(**a**) The location of the AT/G mutation in the UTR of *SETDB2.* Position of the AT/G mutation is on chr13: 50 018 841–50 018 842 (Build Hg19), it is located at −492/−493 before the translation codon ATG of *SETDB2*. (**b**) Sequence showing the *rs386770867* polymorphism.

Sequencing of the 15 exons of *SETDB2* in 10 diploid genomes (five unrelated individuals with atopic disease and five unrelated control individuals) together with a pool of DNA from 32 unrelated individuals resulted in the identification of three mutations. Two missense mutations located in exons 7 and 10 had been genotyped in our previous work (referenced as d8ex7 and d8ex10).^2^ The third variant was an AT/G mutation located in the 5′-UTR of exon 1 and subsequently was designated as *rs386770867* in the NCBI dbSNP database (Figures 1a and b). Previously, we had identified and genotyped 12 SNPs in the *SETDB2* region in an asthma cohort, finding association of IgE with three (*rs7998427, P*=0.02; b1_2, *P*=0.008; *rs11619265*, *P*=0.002).^2^ We therefore genotyped *rs386770867* first in the Australian panel of families, and found that the mutant allele (AT) was significantly associated not only with total serum IgE levels (*P*=0.0012) but also to the phenotype of RASTI (Radio AllergoSorbent Testing Index; the index of the sum of serum IgE levels to the two allergens of house dust mite and grass pollen) (*P*=0.002), both of which are asthma-related phenotypes. The significant association with RASTI was also found in the UK panel (*P*=0.01), making this SNP the most strongly associated with asthma-related traits in *SETDB2* (Table 1).

### Transcription factor binding analysis of *rs386770867*

The sequence spanning *rs386770867* was examined using the transcription factor binding prediction programs TFSearch,^9^ TFScan^10^ and MatInspector.^11^ Three transcription factors (HS$IL6_06, HS$GG_12 [NF-E] and SRY) were predicted to bind the region independently of the mutation. One transcription factor, HS$GMCSF_04 (Ying Yang 1 (YY1)), was predicted to bind the AT allele only, whereas v-Myb was predicted to bind only the G allele (Table 2).

### Transcription factor binding is modulated *in vitro* by *rs386770867*

**Figure 2:**
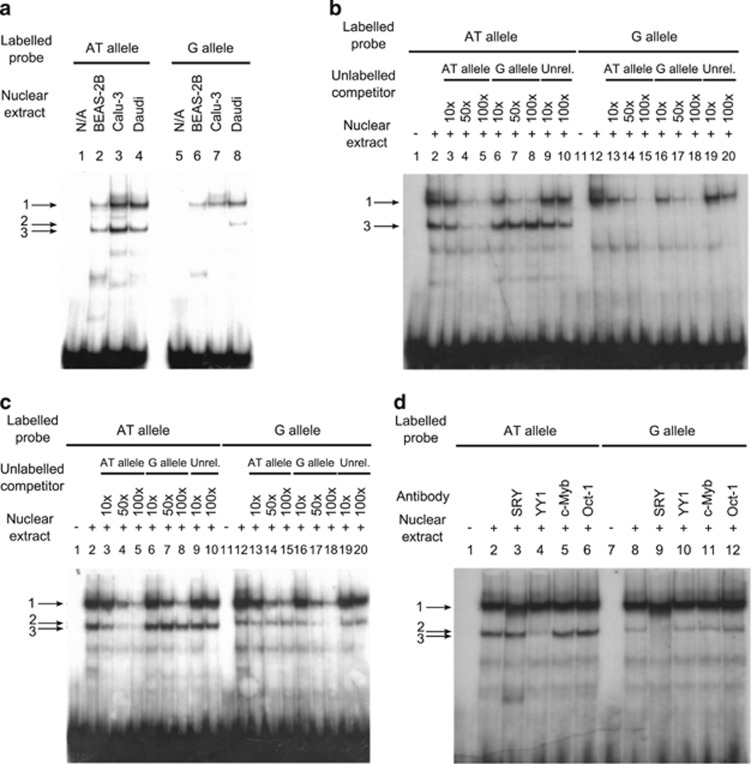
(**a**) EMSA results for *rs386770867* using BEAS-2B, Calu-3 and Daudi nuclear extracts A total of 5 μg of nuclear extract was used per reaction. (**b**) Competition EMSA using Calu-3 nuclear extract. Excess of cold probe used was 10x, 50x and 100x with 5 μg of nuclear extract used per reaction. (**c**) Competition EMSA using 5 μg of Daudi nuclear extract and 10x, 50x and 100x excesses of cold probe. (**d**) Supershift assay using 5 μg of Daudi cell nuclear extract per reaction.

Initial EMSAs using BEAS-2B (bronchial epithelial cell line), Calu-3 (airway epithelial cell line) and Daudi (male B lymphoblast) nuclear extracts identified three major complexes (Figure 2a). Complex 1 was present for both alleles for all the nuclear extracts. Complex 3 formed for the AT allele alone, and again was present for all three nuclear extracts. Complex 2, however, was only observed using the G allele probe and the Daudi nuclear extract. To confirm the specificity of complexes 2 and 3, a series of competition assays were performed using molar excesses of unlabelled probes for each allele, and an unrelated sequence. Competition assays using Calu-3 nuclear extract demonstrated that for both the AT and G probes binding to complex 1 was efficiently competed using unlabelled probes for either allele. The intensity of complex 3 was, however, only reduced in the presence of the unlabelled AT probe (Figure 2b). This was also seen for the Daudi nuclear extract (Figure 2c). Complex 2 was only efficiently competed in the presence of the unlabelled G probe (Figure 2c). The data demonstrated the specific binding of proteins for both AT and G alleles of *rs386770867*.

To identify the proteins responsible for the allele-specific complexes, supershift assays were performed using antibodies for the transcription factors implicated by the bioinformatic analyses of the region: SRY, YY1 and c-Myb. In addition, a reaction using an Oct-1 antibody, not implicated to bind either allele, was included as a negative control. Complex 3 was supershifted in the presence of anti-YY1, whereas complex 2 was abolished by anti-SRY (Figure 2d). Complex 1 was unaffected by any of the antibodies tested.

### Allelotyping

To determine the effect of the allele-specific transcription factor binding on *SETDB2*, the relative expression of the two alleles in heterozygous individuals was investigated by allelotyping.^12, 13^ The 35 heterozygotes for *rs386770867* were identified by genotyping the MRC-A/RNA cohort. For each of the heterozygous individuals, eight replicate matrix-assisted laser desorption/ionization genotyping reactions were performed using both genomic DNA and cDNA for each individual.

The AT:G ratio of peak areas for each replicate genomic DNA result was calculated for all samples. Replicate results outside the 1.5x interquartile range were excluded as outliers (*N*=9), and the mean of the remaining data calculated (mean=1.78; *N*=295). The AT:G peak area ratio in the cDNA of the 35 individuals was compared with the mean ratio from the genomic DNA using a paired-samples *t*-test. This revealed a modest, but highly significant preferential expression of the AT allele across the 35 individuals as a whole (cDNA mean=2.07; gDNA mean=1.78; difference of means=0.286; 95% confidence interval (CI) of the difference=0.232–0.340; fold change=1.16; *P*=1.26 × 10^−21^).

Limiting the analysis to include only those individuals with no outlying replicate cDNA results (*N*=29) confirmed the original finding (cDNA mean=2.10; gDNA mean=1.78; difference of means=0.314; 95% CI of the difference=0.269–0.358; fold change=1.18; *P*=5.66 × 10^−32^) (Table 3). Comparison of each patient separately to the genomic DNA ratio identified 25 individuals with preferential expression of the AT allele, and 1 for the G allele (*P*<0.05) (Table 3). Significant differences were also observed for both sexes when analysed separately, although preferential expression of the AT allele appears to be stronger in males (cDNA mean=2.17; gDNA mean=1.78; difference of means=0.381; 95% CI of the difference=0.306–0.457; fold change=1.21; *P*=5.07 × 10^−18^) compared with that in females (cDNA mean=1.96; gDNA mean=1.78; difference of means=0.171; 95% CI of the difference=0.100–0.242; fold change=1.10; *P*=6.35 × 10^−6^) (Table 3).

### Dual-luciferase reporter assay results

**Figure 3:**
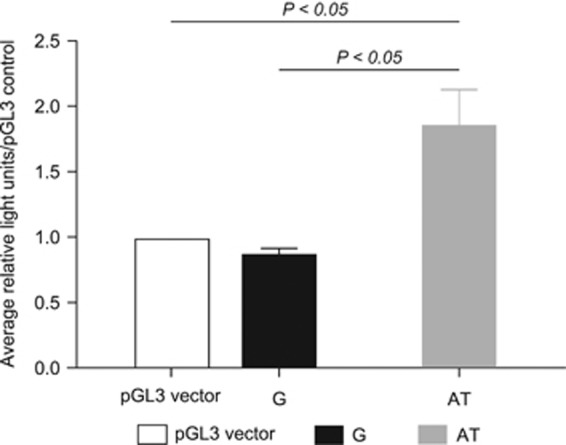
Dual-luciferase reporter assay results of AT and G alleles. The *Renilla* pRL-SV40 plasmid was used as a control for the transfection efficiency. The luciferase expression was documented as average relative light units/pGL3 control.

Jurkat T cells were cultured for the dual-luciferase reporter assay. The *Renilla* pRL-SV40 plasmid was used as a control for the transfection efficiency. The luciferase expression was documented as average relative light units/pGL3 control. The AT allele plasmid showed significantly greater expression levels of luciferase compared with the pGL3 vector *(P*<0.01). The G-allele plasmid showed slightly lower expression of luciferase compared with the pGL3 vector, but this difference was not statistically significant in Jurkat T cells (Figure 3).

## Discussion

Chromosome 13q was first shown to associate with asthma-related traits by whole-genome linkage analysis in 1996.^14^ Subsequently, the genes *PHF11* and *SETDB2* were implicated by positional cloning of the locus.^2, 15^ Our earlier genetic analyses had indicated that variation in total serum IgE levels was attributable, at least in part, to variation in the *PHF11* gene on chromosome 13q14. The coordinate regulation of *PHF11* and the nearby gene *SETDB2* and the possible presence of more distant alleles influencing association suggested, however, that investigation of the function of the locus should include the two genes within this locus.

SETDB2 is a histone H3 lysine 9 methyltransferase that modulates gene expression epigenetically through histone H3 methylation.^16^ Methylation of histone H3 at lysine 9 has emerged as an important player in the formation of heterochromatin, chromatin condensation and transcriptional repression. Depletion of SETDB2 coincides with a loss of CENP proteins and delayed mitosis, suggesting that SETDB2 participates in chromosome condensation and segregation.^16^ Knockdown of *Setdb2* in zebrafish results in a massive expansion of dorsal organizer markers floating head, goosecoid and chordin, as well as a significant increase of fibroblast growth factor 8.^17^ The family of fibroblast growth factors regulates a plethora of developmental processes, including brain patterning, branching morphogenesis and limb development. The recent discovery of the crucial roles of the endocrine-acting fibroblast growth factor 19 subfamily in bile acid, glucose and phosphate homeostasis has sparked renewed interest in the pharmacological potential of this family.^18^

In this report, we first systematically sequenced the 15 exons of *SETDB2* and identified an AT/G mutation in the 5′-UTR (*rs386770867*). The AT allele of this mutation was most highly associated with IgE levels. EMSA analysis of *rs386770867* revealed three main bandshifts, an AT allele-specific complex (band 3), a G-allele-specific complex (band 2) and a complex present for both alleles (band 1). The AT-specific and constant complexes were seen in all three nuclear extracts used (Calu-3, BEAS-2B and Daudi), whereas the G-allele-specific band was only seen in the presence of the Daudi nuclear extract. Supershift experiments indicated that the AT allele complex was due to the YY1 transcription factor binding the probe, whereas the G allele was due to SRY binding. The differences in binding patterns between cell lines may therefore indicate varying regulation of *SETDB2* in different tissue types.

YY1 is a transcription factor, so called because of its ability to act both as an initiator, activator and repressor of transcription.^19, 20^ It is also known as a member of the GLI-Krüppel family of zinc-finger transcription factors.^19^ The protein is highly conserved and ubiquitously expressed. Examples of its role as a repressor are the P5 promoter of adeno-associated virus, c-fos and interleukin-5.^21, 22, 23^ It is believed that YY1 is a repressor by default, but under certain conditions its function can be altered to that of an activator.^24^

An association between YY1 and asthma has already been previously reported. Tumor growth factor-β is an asthma candidate gene, the product of which inhibits B and T cells, decreases IgE production and mast cell proliferation and induces eosinophil apoptosis. Subsequently, it has been shown to be associated with asthma. YY1 has also been shown to inhibit tumor growth factor-β-induced cell differentiation.^25^

*Sry* is the gene required for testis determination and differentiation in mammals.^26^ Recent work has also indicated additional roles for *Sry*, for instance, in the regulation of tyrosine hydroxylase gene transcription.^27^ SRY is sex-specific and so the possibility remains that this could be related to the sex-specific influence on asthma, which has previously been reported. For instance, Weiss *et al.*^28^ reported significant sex-specific differences in FEV_1_ (forced expiratory volume in 1 s), eosinophils and IgE levels, all of which are quantitative traits that are associated with asthma. In addition, two traits, FEV_1_/FVC (forced vital capacity) and eosinophil levels, were found to have sex-specific linkage to regions of the genome and had significant genotype–sex interactions.^28^ Interestingly, our allelotyping data did indicate a stronger preferential expression of the AT allele in males compared with females. This perhaps indicates a greater suppression of allele G expression because of SRY binding in males.

The results of the EMSAs indicate that *rs386770867* changes the sequence from a YY1 binding site to that of SRY. Both transcription factors are able to activate or repress transcription based on the circumstances in which binding takes place. Alleotyping revealed that the AT allele had a modest, but highly significant, increased expression in heterozygous individuals relative to the G allele. Although the scale of the difference was subtle, this could be of biological significance in conditions enhancing IgE production *in vivo*. The dual-luciferase reporter assay showed that the AT allele significantly increases the transcription of luciferase. This therefore indicates that changing from YY1 to SRY binding as a result of the *rs386770867* variant decreases expression of *SETDB2*, consistent with our findings in relation to allele expression in males and females.

Although this work provides additional clarity regarding the mechanisms underlying the chromosome 13q14 asthma locus, many unanswered questions remain. Recent investigation on PHF11 found that it not only regulates the T-cell function^29, 30^ but also is involved in immunoglobulin class switching on B cells.^31^ Therefore, our findings support the role of both genes at this locus influencing asthma-associated traits, although their relative influences are as yet unknown. Future work will need to focus on the functions of PHF11 and SETDB2 in asthma pathophysiology, in particular, how the two molecules work together during immune cell regulation. Additionally, using expression quantitative trait loci data will help to determine both the functional effects of *rs386770867* and the functional role of *SETDB2*. The dissection of regulating elements in the locus will also increase our understanding of the factors that influence the expression of these genes.

## Materials and methods

### *SETDB2* exon sequencing and genotyping of *rs386770867*

Sanger sequencing of 10 diploid genomes (five unrelated individuals with atopic disease and five unrelated control individuals) together with a pool of DNA from 32 unrelated individuals for all 15 exons of *SETDB2* was performed. This gave us 99.9% probability of detecting alleles with a minimum frequency of 0.2 and 99% probability of detecting alleles with a minimum frequency of 0.1. Dilution experiments with known alleles indicated that we were able to detect allele frequencies >0.15 with the pool. An AT/G mutation in the 5′-UTR was identified, subsequently this mutation was designated as *rs386770867*. The mutation was genotyped by a PCR-based restriction fragment length polymorphism method (forward primer: 5′-CGACAGTTCCTCTAGCCG-3′ reverse primer: 5′-CTGAGACAGACAGGCTG**T**A-3′—generating a PCR product of 167/168 bp). The reverse primer contained a modified A to T change (bold) to generate a restriction enzyme site (5′-GTAC-3′) for *Rsa*l, allowing discrimination between the AT and G alleles on a 2.5% agarose gel. The mutation was genotyped in family-based asthma cohorts, which have been described in detail previously.^1^ Briefly, the Australian panel consisted of 364 subjects in 80 nuclear families of European descent from the rural town of Busselton in Western Australia. The families contained a total of 203 offspring forming 172 sib-pairs. The mean age of the children was 12.6±1.3 (s.e.) years, their geometric mean IgE was 55.7±1.1 k Ul^−1^ and their mean skin test index was 4.0±0.41 mm.^14^ The UK panel consisted of 87 nuclear families of British descent recruited when a child of the family attended an asthma clinic in the Oxford region. The families contained 216 offspring (148 sibling pairs) and consisted of 4 large pedigrees and 66 nuclear families. For analysis, three-generation pedigrees were divided into independent two-generation pedigrees, to give a total of 88 nuclear families. The father was atopic in 31 parent pairs, the mother in 17, both parents in 21 and neither parent in 5.^32^ Ethical approvals were given by Australia or the United Kingdom Multicentre Research Ethics Committee. All subjects or their parents gave written informed consent to the study. The genotypes were checked by two experienced researchers. The quantitative trait loci were analysed by the QTL program as described previously.^2^

### Transcription factor binding search programs

Putative transcription factor binding sites in the sequence spanning *rs386770867* were identified using the programs TFSEARCH,^9^ TFSCAN^10^ and MatInspector (Genomatix Software GmbH, Munich, Germany).^11^ Programs were run using the default settings.

### Cell culture and nuclear protein extraction

Daudi (male B lymphoblast), Calu-3 (airway epithelial), BEAS-2B (bronchial epithelial) and Jurkat (T lymphoblast) cell lines were obtained from the American Type Culture Collection (Teddington, UK). Airway epithelium and B and T lymphoblasts are of importance in asthma pathogenesis, making these cell lines relevant for EMSA investigations. In addition, a *SETDB2–PHF11* gene complex has been shown to be expressed in lung tissue and lymphocytes.^2^ Jurkat and Daudi cells were cultured in RPMI 1640 media (Sigma, Gillingham, UK) supplemented to contain final concentrations of 10% fetal bovine serum (Sigma), 10 mmol l^−1^ HEPES (Sigma), 1 mmol l^−1^ sodium pyruvate (Sigma), 4.5 g l^−1^ glucose (Sigma), 2 mmol l^−1^ l-glutamine (Sigma), 20 U ml^−1^ penicillin and 0.1 mg ml^−1^ streptomycin (Sigma). Calu-3 cells were cultured in Dulbecco's modified Eagle's medium (Sigma), supplemented to contain final concentrations of 10% fetal bovine serum, 1 mmol l^−1^ sodium pyruvate, 2 mmol l^−1^ l-glutamine, 20 U ml^−1^ penicillin and 0.1 mg ml^−1^ streptomycin. BEAS-2B cells were cultured in keratinocyte-SFM (serum-free medium) (Gibco-BRL, Paisley, UK) media supplemented with 2 mmol l^−1^ l-glutamine, 5 ng ml^−1^ epidermal growth factor (Gibco-BRL), 50 mg ml^−1^ bovine pituitary extract (Gibco-BRL), 20 U ml^−1^ penicillin and 0.1 mg ml^−1^ streptomycin. All cell lines were cultured at 37 °C and 5% CO_2_. Nuclear extracts were prepared by using a modified Schreiber protocol^33^ and quantified using the Bradford assay.^34^

### Electrophoretic mobility shift assays

Sense and antisense single-stranded oligonucleotides for both alleles of *rs386770867* were designed and synthesized. Each consisted of the SNP allele and 15 bases of 5′ and 3′ flanking sequence. An AGCT tag was added to the 5′ end of each oligonucleotide to facilitate subsequent radiolabelling (*rs386770867* in bold): AT allele: forward, GACTGAGTTTCCAACCTCC**AT**TTCAGCCTGTCTGTC; reverse, GACTGACAGACAGGCTGAA**AT**GGAGGTTGGAAACTC; G allele: forward, GACTGAGTTTCCAACCTCC**G**TTCAGCCTGTCTGTC; reverse, GACTGACAGACAGGCTGAA**C**GGAGGTTGGAAACTC.

The oligonucleotides were annealed to form probes for both alleles and labelled with [α-^32^P]CTP (Amersham Biosciences, Little Chalfont, UK) using the Klenow fragment (New England Biolabs, Hitchin, UK).^35^ Typically, EMSA binding reactions contained 3–5 μg of nuclear extract, 2 μl radiolabelled probe (40 counts per second per μl), 1 × binding buffer (Promega, Hampshire, UK), 0.05–0.1 mg ml^−1^ poly(dIdC)·poly(dI−dC) (Sigma) and 4% glycerol (Sigma) in a total reaction volume of 15 μl. For competition assays, reactions included unlabelled competitor probe at a concentration of 10x, 50x or 100x that of the labelled probe. For supershift assays, 2 μg antibodies were added to the reactions before the first incubation step. The antibodies (Santa Cruz Biotechnology, Santa Cruz, CA, USA) used for supershift assays were YY1, SRY and c-Myb. Reactions were incubated without the labelled probe for 10 min at room temperature. On addition of the labelled probe, reactions were incubated for a further 20 min at room temperature. Products were run on a 6% non-denaturing polyacrylamide gel at 4 °C and 100 V for ~2 h using 0.5x TBE (Tris-borate-EDTA) run buffer before visualization by exposure to Kodak X-Omat AR film (Sigma).

### Allelotyping subjects

Allele-specific expression analysis was performed in a subset of the MRC-A population, MRC-A/RNA. The MRC-A panel consists of 195 siblings and their parents in 95 nuclear pedigrees in which we have previously carried out genome-wide association studies for global gene expression and asthma status.^36, 37^ Our sample contained 357 subjects (183 male) with a mean age in children of 12.2 years (ranging from 2 to 39) and adults of 42 years (ranging from 27 to 61 years). One hundred and thirteen children had doctor diagnosed asthma. Epstein–Barr virus-immortalized lymphoblastoid cell lines were created from blood taken from all the children in the population. The MRC-A/RNA cohort is a subset of the MRC-A population. It is made up of 94 children from the 49 sibling pairs selected from the 200 families on the basis of being the most highly discordant for IgE levels. The Epstein–Barr virus cell lines provided a source of both DNA and RNA from these individuals. Ethical approval was given by the United Kingdom Multicentre Research Ethics Committee. All subjects or their parents gave written informed consent to the study.

### Allelotyping

Allele-specific expression analysis was performed using DNA and RNA from a subset of the MRC-A panel, MRC-A/RNA. Details of the allelotyping method have been described previously.^36^ Briefly, 10 μg of RNA was used to synthesize double-stranded cDNA using the One-cycle cDNA Synthesis Kit (Affymetrix, Santa Clara, CA, USA). Allele peak areas in the Sequenom hME Assay (Sequenom, San Diego, CA, USA) mass spectra are directly proportional to the amount of starting material in the assay. Consequently, the peak areas provide a method of comparing the relative amounts of two alleles for a heterozygote. To determine whether allele-specific expression is occurring, the ratio of the peak areas in the genomic DNA is compared with that in the cDNA to account for any natural bias in peak areas. If the two ratios differ significantly, preferential allele expression is indicated. Amplification was performed in 5 μl reaction volumes with either 5 ng template genomic DNA or cDNA equivalent to 5 ng total RNA. The PCR program was as detailed previously.^38^ Replicate PCRs (*N*=8) for both genomic DNA and cDNA were performed for each subject. PCR products were prepared for mass spectrometry following the standard Sequenom method.^39^

Statistical analyses of the allele ratios for allelotyping were performed using Excel 2010 (Microsoft). For paired-samples *t*-test, unless otherwise stated, the ratio of the peak areas from the mass spectrometry results of allele AT relative to allele G in the cDNA was calculated and compared with the mean ratio in the genomic DNA for all samples to minimize the effect of any potential variation in genomic DNA ratio.

### Dual-luciferase reporter assay

Using the pLG3 promoter plasmid, a construct was made incorporating a 281/280 bp fragment containing *rs386770867*. PCR primers used were: forward, ATAAGCTAGCGAGCCAGGTGAAACCAAGGC; reverse, GCTCGCTCGAGCTTTGACAACCTCAGGTTCC (GCTAGC, *Nhe*l site; CTCGAG, *Xho*l site). PCRs were carried out in individuals homozygous for either the AT or G allele. PCR products were then digested with *Nhe*l and *Xho*l and the bands obtained purified and ligated into the pLG3 promoter plasmid. Clones were picked, cultured overnight for DNA extraction and then sequenced to identify the appropriate constructs.

Jurkat T cells were seeded into 12-well plates at 1x10^6^ cells per ml and 1 μg plasmids were transfected with Lonza Amaxa Cell Line NucleofectorT Kit V (Lonza AG, Castleford, UK). Co-transfection with the *Renilla* pRL-SV40 plasmid (1 μg per transfect) provided a control for transfection efficiency in a dual-luciferase reporter assay system (Promega). The untransfected pGL3 promoter plasmid was used as the negative control. After 24 h transfection, a dual-luciferase assay was carried out and expression of luciferase was measured both according to the manufacturer's protocol. Briefly, the medium was removed from cultured cells and 100 μl 1x Passive Lysis Buffer was added to the wells; then, 25 μl Passive Lysis Buffer lysate was transferred to a new 96-well plate and Luciferase Assay Buffer II was added. Firefly luciferase activity was measured; then, 100 μl Stop & Glo reagent (dual-luciferase reporter assay kit, Promega) was added, and *Renilla* luciferase activity was measured. Three independent experiments were performed and results were analysed using the Student's *t*-tests.

## Figures and Tables

**Table 1:** Details of association between *rs386770867* and the asthma-related traits of IgE level and RASTI

*Polymorphism*	*MAF (AT)*	*Location*	*Position (Build Hg19)*	*AUS1*		*UK1*	
				*LnIgE*	*RASTI*	*LnIgE*	*RASTI*
AT/G	0.42	*SETDB2* 5′-UTR	Chr13: 50 018 841–50 018 842	0.0012	0.0016	0.0963	0.01

Abbreviations: IgE, immunoglobulin E; LnIgE, log_e_ of total serum IgE; MAF, minor allele frequency; UTR, untranslated region; RSATI, Radio Allergo-Sorbent Testing Index; index of specific serum IgE titre against allergens house dust mite and grass pollen using radioallergosorbent test.

**Table 2:** Transcription factors predicted to bind the AT and G alleles of *rs386770867*

*Allele*	*Program*	*Transcription factor*	*Strand*	*Sequence (*5′–3′*)*	*Score*
AT	TFSearch	SRY	−	TGAGTTT	87.3
	TFScan	HS$IL_06	+	TTCC	—
		HS$GMCSF_04	+	C**AT**TT	—
		HS$GG_12	+	CTGTC	—
			+	CTGTC	—
					
G−	TFSearch	SRY	−	TGAGTTT	87.3
	TFScan	HS$GG_12	+	CTGTC	—
			+	CTGTC	—
	MatInspector	v-Myb (Viral?)	−	CTGAA**C**GGAGG	0.915

Searches were performed using the programs TFSearch,^9^ TFScan^10^ and MatInspector.^11^ The polymorphism is shown in boldface in the transcription factor binding sequences. The score is as reported by the search program. No scores are reported by TFScan (indicated by a dash). For MatInspector, the matrix similarity score is given; a value of 1 indicates a perfect match. For TFSearch, a score of 100 indicates a perfect match.

**Table 3:** Paired-samples *t*-test results for individual *rs386770867* heterozygous samples

*Sample*	N	n	*Mean cDNA AT:G*	*Diff of means (cDNA–gDNA)*	*95% CI diff*	*Lower*	*Upper*	*Sig.*	*Overexpressed allele*	*Fold change*
8016_4	8	7	2.7290	0.9447	0.2841	0.661	1.229	0.001	AT	1.5295
8036_3	8	8	2.2489	0.4645	0.1243	0.340	0.589	0.0002	AT	1.2603
8036_4	8	8	2.3455	0.5612	0.6067	−0.046	1.168	0.1127	N/A	1.3145
8046_4	8	8	2.0783	0.2940	0.1860	0.108	0.480	0.0174	AT	1.1648
8052_4	8	7	2.2372	0.4528	0.0483	0.405	0.501	1.677E−06	AT	1.2538
8060_5	8	8	2.0232	0.2389	0.0579	0.181	0.297	8.481E−05	AT	1.1339
8062_4	8	8	1.9644	0.1801	0.1141	0.066	0.294	0.017	AT	1.1009
8067_3	8	8	1.5871	−0.1973	0.1887	−0.386	−0.009	0.091	N/A	0.8895
8080_3	8	8	2.5434	0.7590	0.1593	0.600	0.918	3.347E−05	AT	1.4254
8080_5	8	8	2.1789	0.3946	0.0975	0.297	0.492	9.641E−05	AT	1.2212
8081_4	8	8	2.2138	0.4295	0.1887	0.241	0.618	0.003	AT	1.2407
8100_3	8	8	1.9241	0.1398	0.1342	0.006	0.274	0.080	N/A	1.0784
8103_4	8	6	0.6981	−1.0862	0.0220	−1.108	−1.064	2.228E−09	G	0.3912
8110_4	8	8	2.0563	0.2720	0.1438	0.128	0.416	0.0076	AT	1.1525
8111_5	8	7	2.1026	0.3183	0.0708	0.247	0.389	0.0001	AT	1.1784
8122_4	8	8	1.9307	0.1464	0.1292	0.017	0.276	0.0618	N/A	1.0820
8127_3	8	8	2.0927	0.3084	0.1382	0.170	0.447	0.0033	AT	1.1729
8135_3	8	8	1.9250	0.1407	0.2013	−0.061	0.342	0.2131	N/A	1.0788
8135_4	8	7	1.7092	−0.0751	0.1454	−0.220	0.070	0.3502	N/A	0.9579
8136_3	8	8	2.2603	0.4760	0.1183	0.358	0.594	9.988E−05	AT	1.2667
8146_3	8	8	1.8690	0.0847	0.1456	−0.061	0.230	0.2916174	N/A	1.0475
8146_4	8	8	2.1852	0.4009	0.1167	0.284	0.518	0.0003	AT	1.2247
8148_4	8	8	1.9540	0.1697	0.1202	0.050	0.290	0.0278	AT	1.0951
8148_5	8	8	1.9686	0.1843	0.0895	0.095	0.274	0.0049	AT	1.1033
8154_3	8	8	2.0219	0.2376	0.1158	0.122	0.353	0.0050	AT	1.1331
8158_4	8	8	1.8610	0.0767	0.0561	0.021	0.133	0.0315	AT	1.0430
8176_5	8	7	1.8324	0.0481	1.0002	−0.952	1.048	0.9280	N/A	1.0269
8187_3	8	8	1.8010	0.0167	0.0660	−0.049	0.083	0.6349	N/A	1.0094
8187_4	8	8	1.9886	0.2043	0.1132	0.091	0.318	0.0095	AT	1.1145
8189_3	8	8	2.0520	0.2677	0.1564	0.111	0.424	0.0122	AT	1.1500
8199_3	8	8	2.4459	0.6616	0.2383	0.423	0.900	0.0010	AT	1.3708
8199_4	8	8	2.4312	0.6469	0.1697	0.477	0.817	0.0001	AT	1.3625
8199_5	8	8	2.5618	0.7775	0.0762	0.701	0.854	1.95E−07	AT	1.4357
8208_3	8	8	1.8682	0.0839	0.0907	−0.007	0.175	0.113	N/A	1.0470
8208_4	8	8	2.4574	0.6731	0.3703	0.303	1.043	0.009	AT	1.3772
										
Population	280	273	2.0702	0.2859	0.0538	0.232	0.340	1.258E−21	AT	1.1602
Population (edited)	232	232	2.0979	0.3136	0.0446	0.269	0.358	5.656E−32	AT	1.1757
Male	152	149	2.1657	0.3814	0.0756	0.306	0.457	5.07E−18	AT	1.2137
Male (edited)	128	128	2.1492	0.3649	0.0650	0.300	0.430	3.409E−20	AT	1.2045
Female	128	124	1.9555	0.1712	0.0711	0.100	0.242	6.353E−06	AT	1.0960
Female (edited)	104	104	2.0348	0.2504	0.0571	0.193	0.307	9.278E−14	AT	1.1404

Abbreviations: CI, confidence interval; edited, only cDNA data from samples with no cDNA outliers used; gDNA, genomic DNA; mean gDNA AT:G ratio for population used=1.78431493189407; mean gDNA G:AT ratio for population used=0.567229238822408; *n*, number of replicate results excluding outliers based on lying outside 1.5x interquartile range; *N*, number of replicate results; N/A, not applicable; Sig., significance.

Significance based on paired-samples *t*-test.
