# Erratum: A functional AT/G polymorphism in the 5′-untranslated region of *SETDB2* in the IgE locus on human chromosome 13q14

**DOI:** 10.1038/gene.2016.22

**Published:** 2016-05-12

**Authors:** R J Holt, C Vandiedonck, S A Willis-Owen, J C Knight, W O Cookson, M F Moffatt, Y Zhang

**Keywords:** Disease genetics, Transcription, Asthma

**Correction to:** *Genes and Immunity* (2015) **16**, 488–494; doi:10.1038/gene.2015.36; published online 17 September 2015

**Updated online 12 May 2016:** This article was originally published under a CC BY-NC-SA 4.0 license, but has now been made available under a CC BY 4.0 license. The PDF and HTML versions of the paper have been modified accordingly.

